# In Vivo Assessment of Cold Tolerance through Chlorophyll-*a* Fluorescence in Transgenic Zoysiagrass Expressing Mutant Phytochrome A

**DOI:** 10.1371/journal.pone.0127200

**Published:** 2015-05-26

**Authors:** Mayank Anand Gururani, Jelli Venkatesh, Markkandan Ganesan, Reto Jörg Strasser, Yunjeong Han, Jeong-Il Kim, Hyo-Yeon Lee, Pill-Soon Song

**Affiliations:** 1 Subtropical Horticulture Research Institute, Faculty of Biotechnology, Jeju National University, Jeju 690–756, South Korea; 2 Department of Molecular Biotechnology, Konkuk University, Seoul 143–701, South Korea; 3 Department of Biological Sciences, Presidency University, Kolkata 700073, West Bengal, India; 4 Bioenergetics Laboratory, University of Geneva, Jussy, CH-1254, Geneva, Switzerland; 5 Department of Biotechnology and Kumho Life Science Laboratory, Chonnam National University, Gwangju 500–757, South Korea; 6 School of Biotechnology, Yeungnam University, Gyeongsan, Gyeongbook 712–749, South Korea; University of Hyderabad, INDIA

## Abstract

Chlorophyll-*a* fluorescence analysis provides relevant information about the physiology of plants growing under abiotic stress. In this study, we evaluated the influence of cold stress on the photosynthetic machinery of transgenic turfgrass, *Zoysia japonica*, expressing oat phytochrome A (PhyA) or a hyperactive mutant phytochrome A (S599A) with post-translational phosphorylation blocked. Biochemical analysis of zoysiagrass subjected to cold stress revealed reduced levels of hydrogen peroxide, increased proline accumulation, and enhanced specific activities of antioxidant enzymes compared to those of control plants. Detailed analyses of the chlorophyll-*a* fluorescence data through the so-called OJIP test exhibited a marked difference in the physiological status among transgenic and control plants. Overall, these findings suggest an enhanced level of cold tolerance in S599A zoysiagrass cultivars as reflected in the biochemical and physiological analyses. Further, we propose that chlorophyll-*a* fluorescence analysis using OJIP test is an efficient tool in determining the physiological status of plants under cold stress conditions.

## Introduction

Plants, such as turfgrasses, are constantly subjected to various abiotic stresses such as salinity, heavy metal toxicity, heat/cold, water deficit. The inimical effects of such abiotic stresses often lead to loss in crop yield. In order to improve the inherent stress tolerance, it is imperative to understand the effect of specific stress factors on the photosynthetic machinery of plants. Photosynthetic response of plants under abiotic stress conditions remains largely elusive as it involves complex reactions of several components occurring at various sites of the photosynthetic machinery. To survive under adverse environmental conditions, it is imperative for plants to maintain a balance between the energy supply and consumption via photochemistry and photosynthetic carbon reduction respectively [1,2].

Plants experience cold stress at temperatures above 0°C and below some threshold temperature unique for each species [3]. Cold stress induces several biochemical, physiological, and molecular changes in plant metabolism [4]. Previous studies with Chlorophyll-*a* fluorescence analyses suggest that the primary target for cold stress is located at the reaction center of photosystem II (PSII) [5,6]. Kautsky and Hirsch [7] observed that a dark-adapted leaf when illuminated induces characteristic changes in fluorescence that could be correlated with their photosynthetic rates. The rising phase of fluorescence transient (also known as Kautsky transient) reflects the primary reactions of photosynthesis [8]. With the advent of high-resolution fluorimeters it is possible to record these transients containing useful information on the structural and functional parameters on photosynthetic performance of the organism. When plotted on a logarithmic time scale, the fluorescence rise of the Kautsky transient is polyphasic and can be divided into O-J-I-P steps, where O is the minimal fluorescence (*F*o), P is the peak at about 500 ms (*F*p), and J and I are inflections [9–13]. This test of OJIP transients (the so-called JIP-test) allows us to translate the fluorescence transient measurements into several phenomenological and biophysical parameters that provide information on the PSII functioning of the photosynthetic organism [14–18]. This model compares the photosynthetic activities of stressed and control plants, and it is a non-invasive tool for determining the behavior of the photosynthetic apparatus because the shape of the OJIP fluorescence transient is sensitive to a variety of stress factors on plants [19–24].

Zoysiagrass (*Zoysia japonica*) is an important turfgrass commonly cultivated for home lawns, gardens, sports, and recreational grounds because of its relatively high drought tolerance, disease tolerance, and slow growth [25–26]. Owing to these characteristics, several species of zoysiagrass and bentgrass including their hybrids have been introduced for commercial breeding in the USA [27] and elsewhere. However, because zoysiagrass is a warm-season turfgrass it suffers from cold stress as the grass usually wilts and browns by late autumn. Earlier, we have reported the development of shade-tolerant zoysiagrass cultivar expressing a hyperactive mutant phytochrome A (PhyA) gene, Ser599Ala-PhyA (S599A) [28,29]. Overexpression of S599A in the transgenic zoysiagrass resulted in improved shade tolerance, wider and greener leaves, and short phenotypes with increased number of tillers. Further, a delayed senescence was also noticed in S599A zoysiagrass plants that could be a manifestation of cold tolerance as phytochromes have been suggested to play a vital role in cold tolerance [30,31]. In this study, we have evaluated transgenic zoysiagrass plants carrying hyperactive mutant S599A under cold stress conditions, and demonstrated that the analysis of the recorded OJIP fluorescence transients and its phenomenological and biophysical parameters can be employed for a semi-quantitative assessment of cold tolerance in these plants. In addition, we determined the putative biochemical changes in S599A zoysiagrass subjected to cold stress by estimating the enzyme activities of certain reactive oxygen species (ROS) scavenging enzymes. Our results suggest that overexpression of hyperactive S599A mutant enhances cold stress tolerance in transgenic zoysiagrass, and that the JIP-test is sensitive method to evaluate the response of zoysiagrass plants under cold stress.

## Materials and Methods

### Plant materials and growth conditions

Two independent transgenic lines of zoysiagrass (*Z*. *japonica*), one expressing the oat PhyA and the other expressing a hyperactive mutant (S599A) generated via *Agrobacterium*-mediated transformation as described earlier [28], were used for the present study. Non-transgenic zoysiagrass plants (NT) were used as control. Two independent transformants (S599A-2-14 and S599A-2-18) were selected for S599A zoysiagrass plants. Approximately 2-year-old zoysiagrass transgenic and control plants obtained after three growth seasons (April 2010 to December 2012) were used. Plants grown in pots (15 cm in diameter) containing sterile biopeat were maintained in a greenhouse at 22 ± 2°C with 16:8-h light:dark regime.

### Cold stress treatment

To evaluate the performance under cold conditions, transgenic zoysiagrass lines grown in pots were maintained in a plant growth chamber at 10°C for 2 weeks under 16:8-h light:dark regime. Three pots representing each transgenic and NT lines were kept in cold chamber for stress tolerance assessment. Similarly, three pots each representing transgenic and NT lines were maintained in the greenhouse (at 25°C under 16:8-h light:dark regime) that served as controls. Chlorophyll-*a* fluorescence measurements were done in dark-adapted conditions of cold stressed and control plants after 2 weeks. Subsequently, leaves from cold treated plants were sampled and frozen in liquid nitrogen and stored at −80°C for biochemical analyses.

### Estimation of hydrogen peroxide and Proline levels

Hydrogen peroxide (H_2_O_2_) content in leaves collected from the NT, PhyA, S599A-2-14, and S599A-2-18 zoysiagrass plants subjected to cold stress was determined by using the ferrous ammonium sulfate/xylenol orange method [32]. A standard curve was prepared from serial dilutions (0 to 5 μM concentration of H_2_O_2_ and *R* ^*2*^ coefficient of 0.9403) to calculate H_2_O_2_ concentrations. Data were normalized and expressed in μmol H_2_O_2_ g^−1^ fresh weight of leaves.

Proline extraction and colorimetric estimation were carried out in leaf samples from NT, PhyA, S599A-2-14, and S599A-2-18 zoysiagrass plants subjected to cold stress, as described earlier [33]. Approximately 500 mg of leaf samples, ground in mortar and pestle in the presence of liquid N_2_ were homogenized in 10 mL of 3% aqueous sulfosalicylic acid. Equal volumes of the filtered homogenate, acid-ninhydrin, and glacial acetic acid were mixed and allowed to react for 1 h in a boiling water bath. After 1 h, tubes were placed on ice to terminate the reaction followed by extraction with 4 ml toluene. The chromophore-containing layer was brought to room temperature and absorbance was recorded at 520 nm. Proline concentration was determined from a standard curve and calculated on a fresh weight basis (μmol proline g^−1^ fresh weight).

### Estimation of ROS-scavenging enzyme activity

Leaf samples from the NT, PhyA, S599A-2-14, and S599A-2-18 zoysiagrass plants, subjected to cold stress, were pulverized in a chilled mortar-pestle in the presence of liquid N_2_. Protein was extracted with a buffer containing 0.2 M potassium phosphate buffer (pH 7.5), 50% (v/v) glycerol, 16 mM MgSO_4_, 0.2 mM phenyl methyl sulfonyl fluoride, and 0.2% polyvinylpolypyrrolidone and centrifuged at 13,000 × *g* for 30 min at 4°C. The supernatant was collected, and the protein content was determined, as described earlier (Bradford, 1976). Specific enzyme activities for ascorbate peroxidase (APx), catalase (CAT), superoxide dismutase (SOD), and glutathione reductase (GR) were determined following the methods described earlier [34–36].

### Chlorophyll-a fluorescence measurements

**Fig 1:**
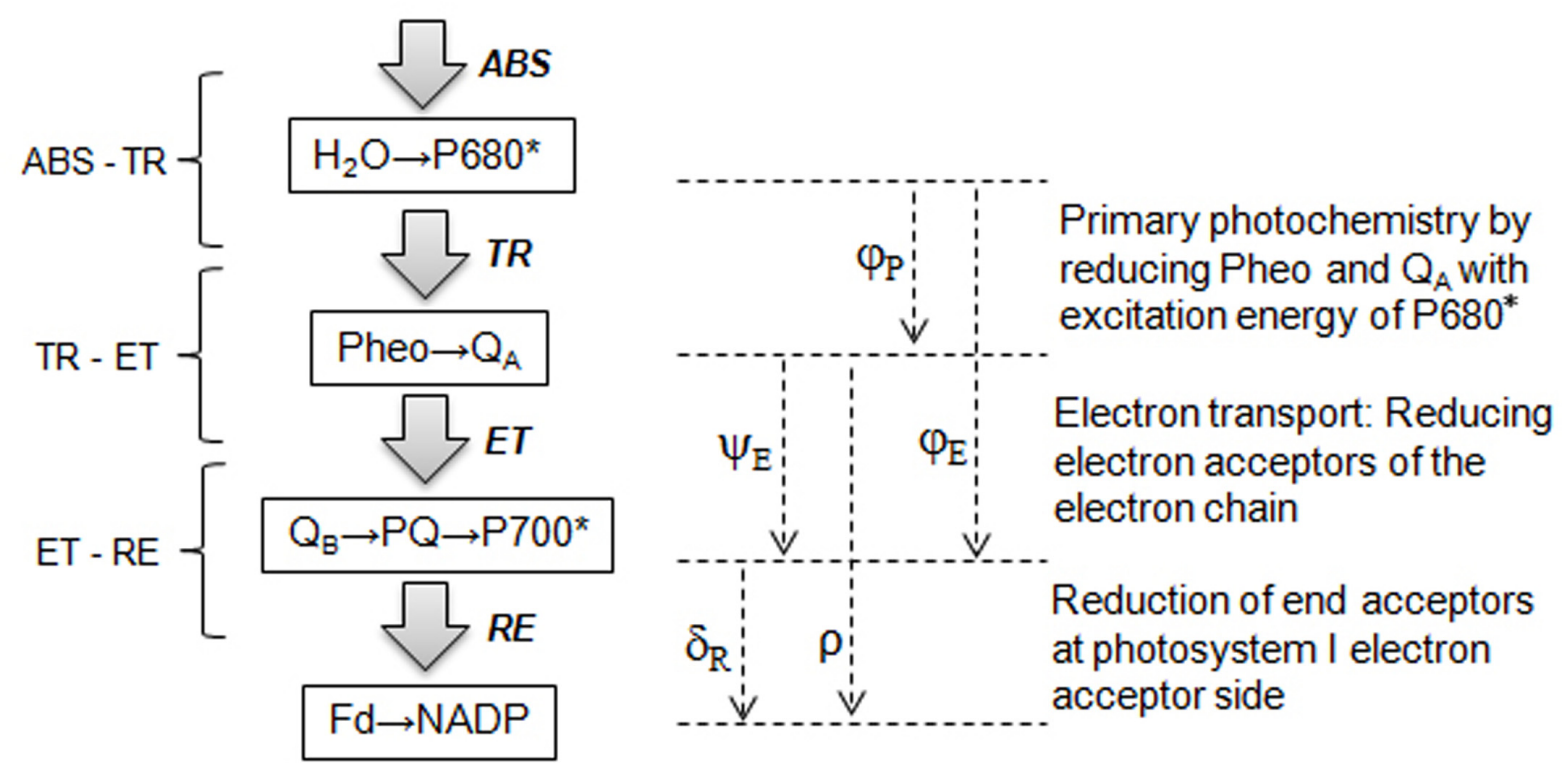
Schematic representation of the JIP-test expressed in terms of sequential energy fluxes from absorbance (ABS) of photons by PSII antenna, intermediate trapping flux (TR), electron transport (ET) and the reduction of the end-electron acceptors at the PS I electron acceptor side (RE) driven by PS I. Formulae and glossary of terms used by the JIP-test are presented in Table 1.

Pots containing transgenic and NT control plants subjected to cold stress were dark-adapted for 1 h prior to taking Chlorophyll-*a* fluorescence measurements. Measurements were made on fully expanded leaves of three plants of each line under dark conditions with a pocket PEA (Plant Efficiency Analyser, Hansatech Instruments Ltd., King’s Lynn, Norfolk, UK). Chlorophyll-*a* fluorescence readings from transgenic and NT plants maintained in greenhouse conditions were used as controls. An actinic light of 3000 μmol photons m^−2^ s^−1^ was used for the determination of fluorescence induction, and fluorescence was recorded at wavelengths around 685 nm. Five random leaves were used per pot from each treatment in triplicates. The maximal fluorescence (*F*_M_) and the minimal fluorescence (*F*_0_) of sampled leaves were used to calculate the *F*_v_/*F*_M_ ratio, which is related to quantum yield of PSII photochemistry (see e.g., [37]). The data were analyzed using Biolyzer software program that calculates according to the so-called JIP-test equations [38–39]. The JIP-test equations are based on theory of energy fluxes in biomembranes [40], which supports the general derivation for the actual quantum yield of primary photochemistry φ_Px_ = TR_x_/ABS = 1 − *F*_x_/*F*_M_, according to Paillotin [41]. These equations explain that each energy flux of the energy cascade from the photon absorption flux (ABS) is converted into a free energy flux (RE), transported via electron transport (ET), and stored by the reduction of end-electron acceptors of photosystem I (PSI) (**Fig 1)**. The formulae and definitions of terms used in the JIP-test are listed in **Table 1**. The following fluorescence intensity values were used from the original measurements representing the subsequent steps: minimal fluorescence intensity at 20 μs, when all PS II reaction centers (RCs) are open (the O step); fluorescence intensity at 300 μs; fluorescence intensity at 2 ms (the J step); fluorescence intensity at 30 ms (the I step); and the maximal intensity when all PS II RCs are closed (the P step).

**Table 1:** Formulae and glossary of terms used by the JIP-test (modified after Strasser et al. 2004).

**Data extracted from the recorded fluorescence transient OJIP**	
F_t_	fluorescence at time t after onset of actinic illumination
F_50μs_ or F_20μs_	minimal reliable recorded fluorescence, at 50 μs with the PEA- or 20 μs with the Handy-PEA-fluorimeter
F_300μs_	fluorescence intensity at 300μs
F_J_ ≡ F_2ms_	fluorescence intensity at the J-step (2 ms) of OJIP
F_I_ ≡ F_30ms_	fluorescence intensity at the I-step (30 ms) of OJIP
F_P_	maximal recorded fluorescence intensity, at the peak P of OJIP
t_FM_	time (ms) to reach the maximal fluorescence intensity F_M_
Area	total complementary area between the fluorescence induction curve and F = F_M_
**Fluorescence parameters derived from the extracted data**	
F_0_ ≅ F_50μs_ or ≅ F_20μs_	minimal fluorescence (all PSII RCs are assumed to be open)
F_M_ (= F_P_)	maximal fluorescence, when all PSII RCs are closed (equal to F_P_ when the actinic light intensity is above 500 μmol photons m^-2^ s^-1^ and provided that all RCs are active as Q_A_ reducing)
F_υ_≡ F_t_-F_0_	variable fluorescence at time t
F_V_ ≡ F_M_-F_0_	maximal variable fluorescence
V_t_ ≡ F_υ_/F_V_ ≡ (F_t_-F_0_)/(F_M_-F_0_)	relative variable fluorescence at time t
M_0_ ≡ [(ΔF/Δt)_0_]/(F_M_-F_50μs_) ≡ 4 (F_300μs_-F_50μs_)/(F_M_- F_50μs_)	approximated initial slope (in ms^-1^) of the fluorescence transient normalised on the maximal variable fluorescence F_V_
**Specific energy fluxes (per Q**_**A**_ **-reducing PSII reaction center—RC)**	
ABS /RC = M_0_ (1/V_J_)(1/φ_Po_)	absorption flux (of antenna Chls) per RC
TR_0_/RC = M_0_ (1/V_J_)	trapped energy flux (leading to Q_A_ reduction) per RC
ET_0_/ RC = M_0_ (1/V_J_)ψ_Eo_	electron transport flux (further than Q_A_^⚿^) per RC
RE_0_/RC = M_0_ (1/V_J_)ψ_Eo_ δ_Ro_	electron flux reducing end electron acceptors at the PSI acceptor side, per RC
**Quantum yields and efficiencies**	
φ_Pt_ ≡ TR_t_/ABS = [1-(F_t_/F_M_)] = ΔF_t_/F_M_	quantum yield for primary photochemistry at any time t, according to the general equation of Paillotin (1976)
φ_Po_ ≡ TR_0_/ABS = [1-(F_0_/F_M_)]	maximum quantum yield for primary photochemistry
ψ_Eo_ ≡ ET_0_/TR_0_ = (1-V_J_)	efficiency/probability for electron transport (ET), i.e. efficiency/probability that an electron moves further than Q_A_^⚿^
φ_Eo_ ≡ ET_0_/ABS = [1-(F_0_/F_M_)]ψ_Eo_	quantum yield for electron transport (ET)
δ_Ro_ ≡ RE_0_/ET_0_ = (1-V_I_)/(1-V_J_)	efficiency/probability with which an electron from the intersystem electron carriers moves to reduce end electron acceptors at the PSI acceptor side (RE)
φ_Ro_ ≡ RE_0_/ABS = [1-(F_0_/F_M_)]ψ_Eo_ δ_Ro_	quantum yield for reduction of end electron acceptors at the PSI acceptor side (RE)
γ_RC_ = Chl_RC_/Chl_total_ = RC/(ABS+RC)	probability that a PSII Chl molecule functions as RC
RC/ABS = γ_RC_/(1-γ_RC_) = φ_Po_ (V_J_/ M_0_)	Q_A_-reducing RCs per PSII antenna Chl (reciprocal of ABS/RC)
**Phenomenological fluxes**	
ABS/CS = *F*o or ABS/CSM = *F*_M_	absorption per excited cross-section
TRo/CS = ΦPo·(ABS/CS)	trapping per excited cross-section
ETo/CS = ΦPo·Ψo·(ABS/CS)	electron transport per excited cross-section
**Performance indexes**	
PI_ABS_ ≡ [γ_RC_/(1-γ_RC_)]. [φ_Po_/(1- φ_Po_)].[ψ_o_/(1- ψ_o_]	performance index (potential) for energy conservation from exciton to the reduction of intersystem electron acceptors
PI_total_ ≡ (PI_ABS_).(δ_Ro_/1- δ_Ro_)	performance index (potential) for energy conservation from exciton to the reduction of PSI end acceptors

Chlorophyll-*a* fluorescence transients recorded in the dark-adapted zoysiagrass samples under cold stress were further analyzed by the JIP-test to obtain information on several structural and functional parameters that indirectly quantify the photosynthetic behavior of the experimental plant samples. The radar plot thus illustrates the calculated average values of the photosynthetic parameters of the NT, PhyA, and the S599A lines (S599A-2-14 and S599A-2-18) of zoysiagrass.

## Results

### S599A zoysiagrass accumulated less hydrogen peroxide and high proline

**Fig 2:**
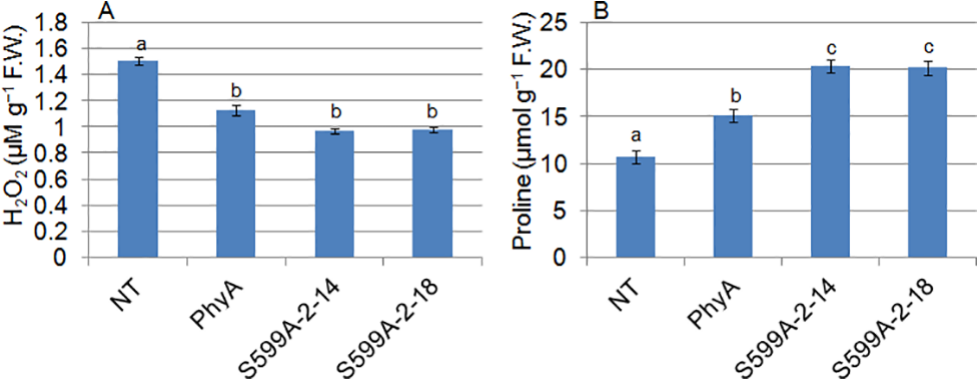
Estimation of (A) hydrogen peroxide content and (B) proline content in non-transgenic control (NT), plants expressing oat PhyA (PhyA) and plants expressing mutant PhyA (S599A-2-14 and S599A-2-18) subjected to cold stress. Different letters in each column indicate significant differences (*p≤0*.*05*) in between treatments after Tukey’s test (n = 3).

The cold stressed transgenic zoysiagrass, expressing hyperactive S599A mutant lines, S599A-2-14 and S599A-2-18, showed significantly lesser H_2_O_2_ accumulation than NT control plants (**Fig 2A)**. Similarly, the PhyA plants also accumulated lower H_2_O_2_ than the NT control plants. On average, the NT plants accumulated 54.6 and 33.3% higher levels of H_2_O_2_ than the S599A and PhyA zoysiagrass lines, respectively. Further, difference in the H_2_O_2_ content between S599A and PhyA lines was found to be non-significant (at *P* ≤ 0.05) after comparison of the means in nine replicates of each line from three independent experiments.

Estimation of proline concentration in NT and transgenic zoysiagrass plants, expressing PhyA or S599A showed a marked increase in transgenic lines compared to NT control lines (**Fig 2B**). The PhyA lines had approximately 42% increased proline accumulation while zoysiagrass lines, expressing S599A showed around 88% increased proline levels compared to the NT control lines. Comparison of the means of nine replicates from of each line from three independent experiments showed a significant difference (at *P* ≤ 0.05) between the proline accumulation among PhyA, S599A, and NT control lines. Reduced levels of H_2_O_2_ and increased proline accumulation indicate that zoysiagrass lines overexpressing oat PhyA or S599A are more tolerant to cold temperature than the NT lines.

### S599A zoysiagrass exhibits higher activity of ROS-scavenging enzymes

**Fig 3:**
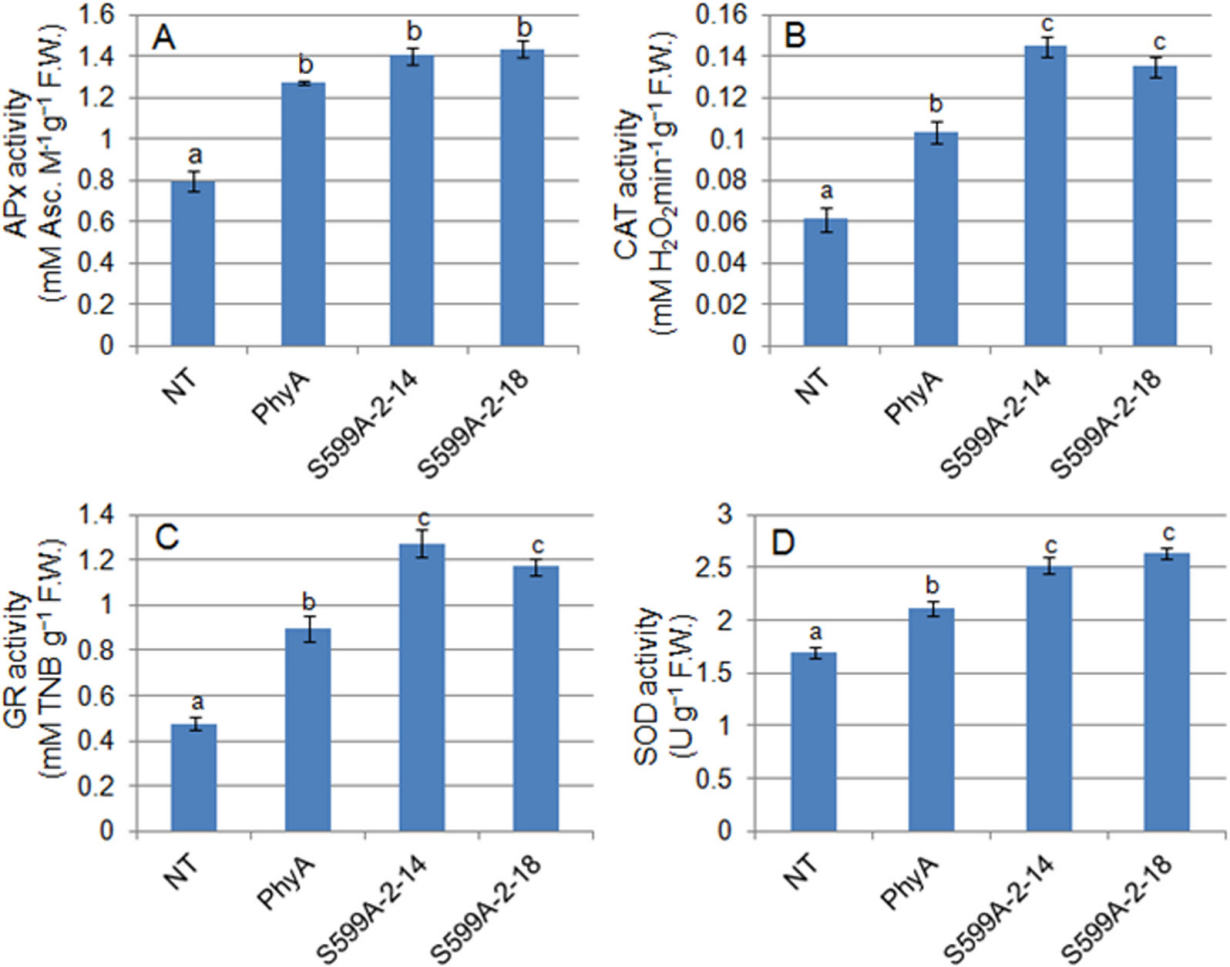
Estimation of ROS-scavenging enzymes activities. Specific enzyme activities of (A) APx, (B) CAT, (C) GR and (D) SOD were determined in the leaves of in non-transgenic control (NT) plants, expressing oat PhyA (PhyA) and plants expressing mutant PhyA (S599A-2-14 and S599A-2-18) subjected to cold stress. Different letters in each column indicate significant differences (*p≤0*.*05*) in between treatments after Tukey’s test (n = 5).

We have measured and analyzed specific activity of ROS-scavenging enzymes in PhyA, S599A, and NT zoysiagrass plants subjected to cold stress. The specific enzyme activity of APx was 1.6-fold and 1.7-fold higher in PhyA and S599A (S599A-2-14 and S599A-2-18) plants respectively, than in the NT control plants (**Fig 3A)**. CAT specific activity was 1.6-fold higher in PhyA plants than in NT control plants, while S599A plants had 2.1 to 2.3-fold higher CAT activity than that in NT control plants (**Fig 3B)**. Similar trends were observed with GR where PhyA plants showed approximately 1.9-fold, while S599A plants showed 2.4 to 2.7-fold increased GR activity than that in NT control plants (**Fig 3C)**. The lowest fold increase was observed in the specific activity of SOD where PhyA plants exhibited 1.3-fold, while S599A plants had 1.5 to 1.6-fold increased SOD activities (**Fig 3D)**.

### S599A zoysiagrass plants were photosynthetically more efficient under cold stress

**Fig 4:**
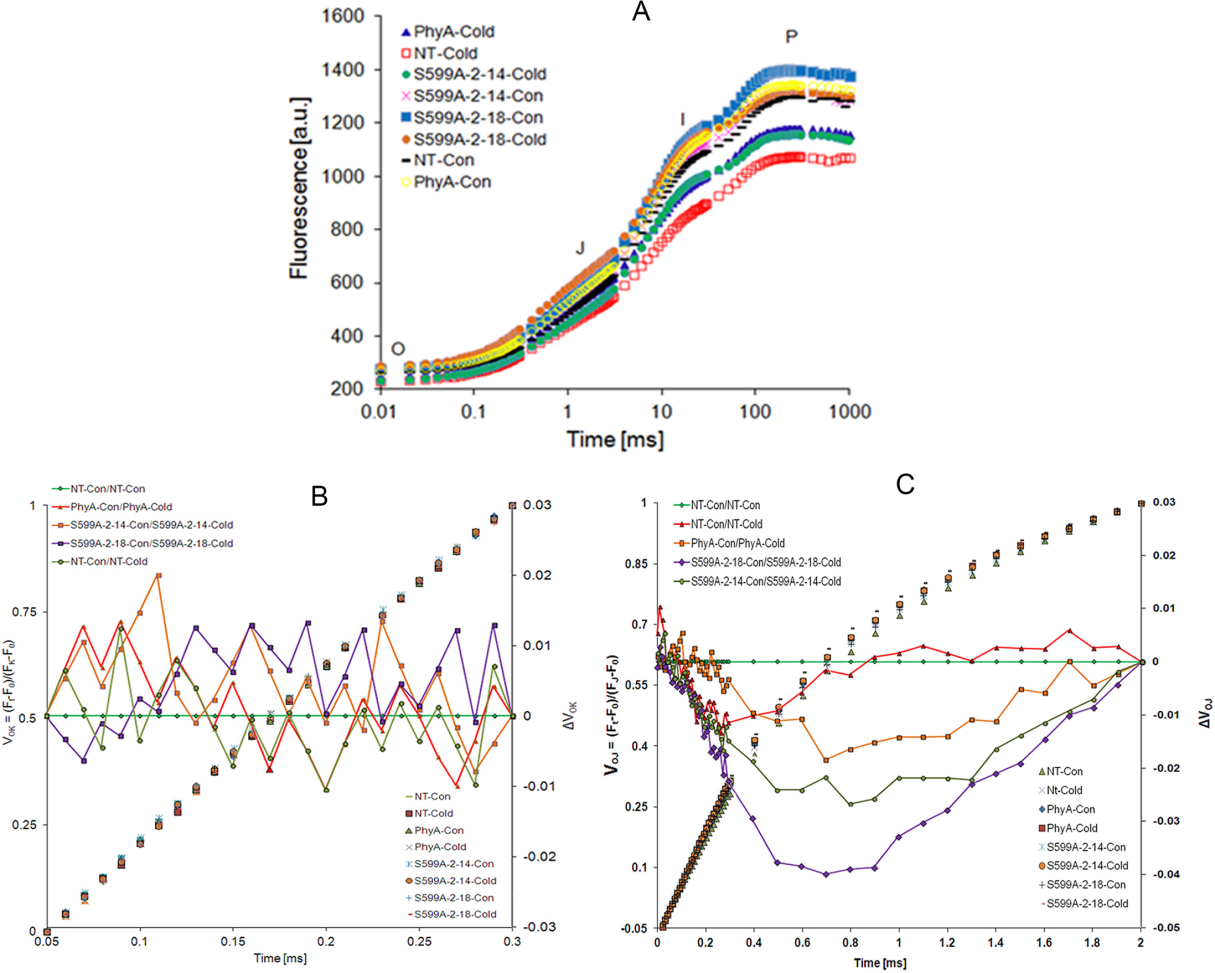
**A.** Chlorophyll-a fluorescence kinetics of the OJIP curve of dark-adapted leaves of zoysiagrass plants. Transient curves of each line represent the average of 15 measurements per treatment. PhyA-Cold, plants expressing oat PhyA subjected to cold stress; NT-Cold, non-transgenic plants subjected to cold stress; S599A-2-14-Cold, transgenic plants expressing mutant PhyA line-14 subjected to cold stress; S599A-2-14-Con, transgenic plants expressing mutant PhyA line-14 under normal conditions; S599A-2-18-Con, transgenic plants expressing mutant PhyA line-18 under normal conditions; S599A-2-18-Cold, transgenic plants expressing mutant PhyA line-18 subjected to cold stress; NT-Con, non-transgenic plants under normal conditions; PhyA-Con, plants expressing oat PhyA under normal conditions. Fluorescence transients of NT-C, PhyA-C, and S599A (S599A-2-14-C and S599A-2-18-C) plants under cold stress conditions and their respective control plants (NT, PhyA, S599A-2-14, and S599A-2-18) maintained under greenhouse conditions are plotted with black dashes, blue triangles, green circles, blue squares, yellow circles, red squares, pink crosses, and brown circles, respectively. **B.** The L-band: Relative variable fluorescence V_OK_ = (F_t_ − F_o_)/(F_k_ − F_o_) and differences between NT, PhyA and S599A plants under normal and cold stress conditions. Curves without lines (left axis) and curves with solid symbols represent difference kinetics, ΔV_OK_ of plants under normal and cold stress conditions on the logarithmic scale (right vertical axis). **C.** The K-band; Double normalization of the data for NT, PhyA and S599A plants under normal and cold stress conditions at F_0_ and F_J_, V_OJ_ = (F_t_–F_0_)/(F_J_–F_0_). NT-Con/NT-Con as green diamonds; NT-Con/NT-Cold as red triangles; PhyA-Con/PhyA-Cold as brown squares; S599A-2-18-Con/S599A-2-18-Cold as purple diamonds and S599A-2-14-Con/S599A-2-14-Cold as green circles.

To determine the photosynthetic performance of NT, PhyA, and S599A zoysiagrass under cold stress, fluorescence JIP-test was used. Chlorophyll-*a* fluorescence transients of dark-adapted leaves of zoysiagrass are shown on a logarithmic scale from 20 μs to 1 s in **Fig 4A**. A typical OJIP shape was found in all the samples with similar maximum variable fluorescence (*F*_M_ − *F*_0_ = *F*_V_), illustrating that all the plants were almost equally photosynthetically active.

### The L-band

To further evaluate differences between NT, PhyA, and S599A lines, fluorescence data were normalized between O (50 μs) and K (300 μs) steps, as *V*_OK_ = (*F*_t_ − *F*_0_)/(*F*_K_ − *F*_0_), and plotted as difference kinetics Δ*V*_OK_ = *V*_OK(transgenic)_ − *V*_OK (control)_ in the time range of 50–300 μs revealing the L-band (**Fig 4B)**. The L-band has been suggested to be an indicator of the energetic connectivity (grouping) of the PSII units. Subtraction of the data of NT leaf samples from PhyA and S599A leaf samples exhibited negative L-bands indicating a higher cooperativity of excitation energy exchange between PSII units relative to the reference sample. A higher cooperativity results in efficient consumption of the excitation energy and a higher stability of the system [40,42]. Phenomenologically, this cooperativity reflects in a sigmoidal rise between 0 and 300 μs that parallels a maximal decrease in Δ*V*_OK_ at about 150 μs, thus resulting in a decrease of L-band.

### The K-band

Furthermore, fluorescence data were normalized between the steps O and J (2 ms), as *V*_OJ_ = (*F*_t_ − *F*_0_)/(*F*_J_ − *F*_0_), and plotted with the difference kinetics Δ*V*_OJ_ between the samples relatively to their controls in the 0–300 μs range revealing the K-band (**Fig 4C)**. An increased slope (positive K-band) indicates an increased reduction rate of quinone (*Q*_A_), the primary electron acceptor of PSII, from *Q*_A_ to *Q*_A_^−^, which could mean that the oxygen evolving complex (OEC) becomes leaky and offers access to non-water electron donors [13]. A positive K-band (at about 300 μs) suggests that the OEC is either inactivated or there is an increase in the functional PSII antenna size [43].

### Radar plot

**Fig 5:**
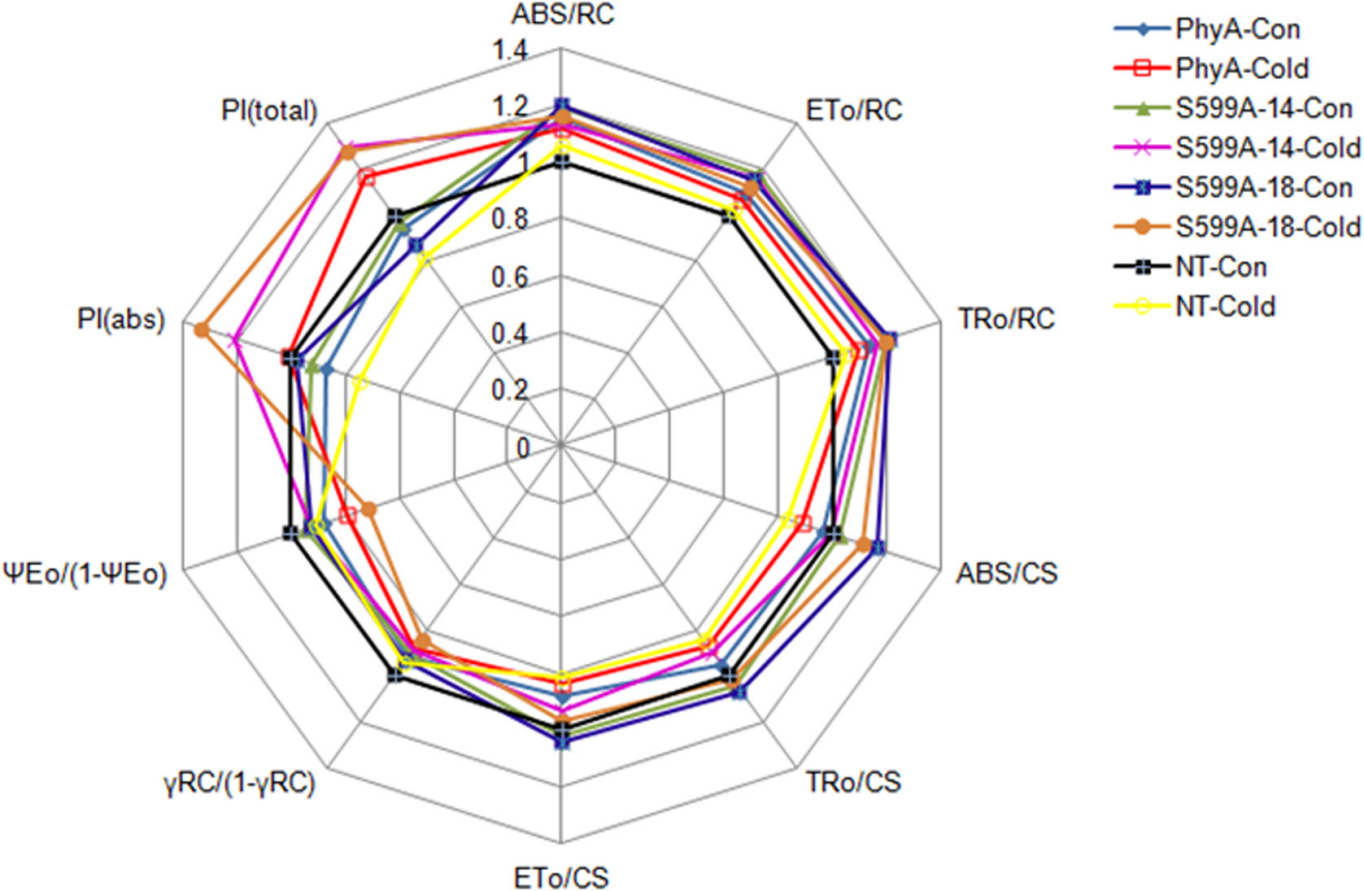
Radar plot with a series of parameters derived from JIP-test analyses of the experimental fluorescence OJIP transients. This plot depicts possible differences in the structure and function of the photosynthetic apparatus under different conditions; PhyA plants under normal conditions (PhyA-Con) as closed blue diamonds, PhyA plants under cold stress (PhyA-Cold) as open red squares, S599A-2-14 plants under normal conditions (S599A-2-14-Con) as closed green triangles, S599A-2-14 plants under cold stress (S599A-2-14-Cold) as pink crosses, S599A-2-18 plants under normal conditions (S599A-2-18-Con) as closed blue squares, S599A-2-18 plants under cold stress (S599A-2-18-Cold) as closed brown circles, non-transgenic plants under normal conditions (NT-Con) as closed black squares and non-transgenic plants under cold stress (NT-Cold) are depicted as open yellow circles. The deviation of the behavior pattern from the regular polygon demonstrates the fractional impact, compared to the untreated plants of the corresponding treatment. Details of each parameter are discussed in the Results section.

Relative values of ten major JIP-test parameters are shown in a radar plot (**Fig 5)**. The parameter ABS/RC reflects total absorption of PSII antenna chlorophylls per active RC. JIP-analyses of PhyA, S599A, and NT control zoysiagrass lines also revealed that overexpression of PhyA or S599A significantly increased the absorption flux of photons per active RCs. However, we note that a concomitant increase in ABS/RC only means an increase in the apparent antenna size and does not mean a structural increase in the antenna size of a biochemical complex. Electron transport to the intersystem chain per active PSII-RC is represented here by the parameter ET_0_/RC. Higher ET_0_/RC in PhyA and S599A zoysiagrass lines indicated a thermal activation of the dark reactions, as described earlier [38]. The rate at which an exciton is trapped by an active RC is expressed by the parameter TR/RC. Trapping of an exciton by the RC results in the reduction of *Q*_A_ to *Q*_A_^−^ and since at time zero, all the RCs are open, the maximal value of TR/RC can be expressed as TR_0_/RC. Similar to ABS/RC and ET_0_/RC, a higher value of TR_0_/RC in PhyA and S599A lines was observed. The phenomenological energy fluxes (per excited cross-section, CS) for absorption (ABS/CS), trapping (TRo/CS), and electron transport (ETo/CS) were also calculated. The value of all the three phenomenological energy fluxes was reduced in all zoysiagrass lines when the plants were subjected to cold stress. The PhyA lines recorded the largest difference in ABS/CS values of samples under normal and cold stress conditions, while the S599A-2-14 lines recorded the largest difference in TRo/CS and ETo/CS values of samples under normal and cold stress conditions. The parameter γ_RC_/(1−γ_RC_) or RC/ABS expresses the fraction of PS II chlorophyll-*a* molecules that function as RCs (**Table 1**). Thus, γ_RC_/(1−γ_RC_) is proportional to the fraction of absorbed energy by excitation that reaches the RCs. In all the plant lines, the value of γ_RC_/(1−γ_RC_) was reduced when the plants were subjected to cold stress. The parameter ψ_0_/(1 − ψ_0_) represents the efficiency with which a trapped exciton transfers an electron to the photosynthetic ET chain further than *Q*_A_^−^. The cold stress-induced loss of ET capacity was indicated by a decrease in the value of ψ_0_/(1 − ψ_0_) in all the zoysiagrass lines. The multi-parametric expression, the photosynthetic performance index (PI_ABS_), represents a combination of three independent steps, ABS, TR, and ET that contribute to overall photosynthesis [14], while the performance index PI_total_ has been suggested to represent partial potentials for energy conservation. PI_total_ is, therefore, closely related to plant’s overall growth and survival under stress conditions. The PhyA as well as the S599A lines exhibited an increase in PI_ABS_ and PI_total_ after the plants were exposed to cold stress.

## Discussion

**Fig 6:**
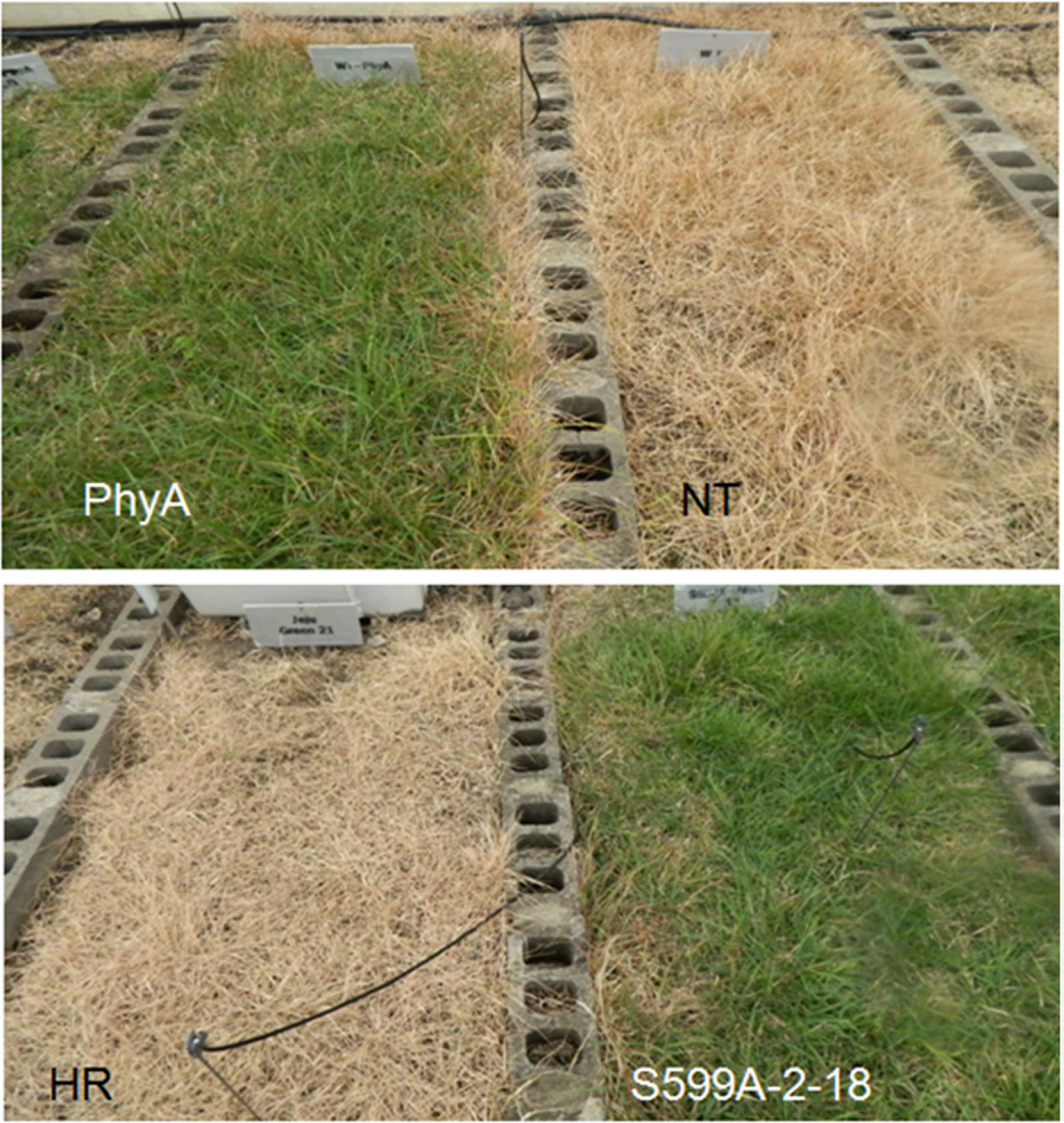
Phenotypic differences between non-transgenic (NT) and transgenic plants expressing wild-type oat PhyA (PhyA) in the above panel and control plants carrying only vector with a *BAR* gene for herbicide resistance (HR) and transgenic plants expressing mutant PhyA (S999A-PhyA-18) in the lower panel. This photograph was taken in late October 2014 at Jeju National University experimental plots, Jeju, South Korea.

Several reports have described the response of seed germination to high/low temperatures that are mediated by PhyA, PhyB, or PhyE in *Arabidopsis* plants [29,44,45]. Similarly, PhyA, PhyB, and PhyD have been demonstrated as crucial factors for breaking seed dormancy induced by low temperature [46]. In a previous study, we have reported the development of transgenic zoysiagrass expressing wild-type oat PhyA (PhyA) or a hyperactive mutant gene (S599A). S599A plants express a mutant oat PhyA in which a phosphorylation site involved in light-signal attenuation was blocked [47]. Further, in addition to phenotypic changes and improved traits reported earlier [28], S599A plants were shown to survive in adverse environmental conditions such as low temperature both *in vitro* and in the field. In the experimental fields, we consistently noticed delayed senescence and later browning in PhyA and S599A plants at the start of the winter season when compared to the NT and herbicide resistant (HR, transgenic zoysiagrass expressing *bar* gene for herbicide resistance as a selectable marker) plants (**Fig 6)**. Since the HR and NT plants showed similar responses to cold stress in terms of photosynthetic efficiency and biochemical activities (data not shown), only one of these two lines i.e. NT was taken as control for the present analysis.

Abiotic stress caused by cold conditions results in an overreduction of the ET chain that consequently leads to photooxidation [1,48]. Under these conditions, electrons are allocated to dioxygen (O_2_) which is then used in the Mehler peroxidase reaction. In this reaction, O_2_ is reduced to superoxide (O_2_^−^) and the O_2_^−^ to H_2_O_2_, both of which are potent ROS molecules. H_2_O_2_ as a potent inhibitor of photosynthesis can severely hamper the CO_2_ fixation by oxidizing the thiol-modulated enzymes of the Calvin-Benson cycle [49]. Over-accumulation of H_2_O_2_ in plants under various abiotic stresses is a common phenomenon. We recorded significantly reduced levels of H_2_O_2_ in cold stressed PhyA and S599A zoysiagrass lines compared to that in NT lines, indicating that transgenic zoysiagrass lines sustained the cold stress considerably better than the NT lines (**Fig 2A)**. Several studies have demonstrated induction of enhanced H_2_O_2_ accumulation during cold stress in higher plants [50–53]. Enhanced levels of proline accumulation in plants exposed to various abiotic stresses is well documented [54,55]. Besides playing the role of a ROS-scavenger, proline has also been suggested as a non-water electron donor to PSII during abiotic stress conditions in plants [54,56]. Increased proline accumulation in PhyA and S599A zoysiagrass plants **(Fig 2B)** clearly indicates that transgenic plants perform better under cold stress conditions and demonstrates the requirement of proline in the integrity of the cellular system for survival under stress conditions. Overproduction of ROS in plants under stress is counteracted by the ROS-scavenging enzymes. Our data suggests that expression of oat PhyA or oat S599A in zoysiagrass plants increases specific enzyme activities of APx, CAT, GR, and SOD (**Fig 3)** that facilitates efficient removal of free radicals produced under cold stress conditions. Enhanced activity of major ROS-scavenging enzymes in rice plants tolerant to cold stress has been reported earlier [57]. Therefore, in agreement with the earlier studies [33,56], we infer that the increased proline accumulation and higher activity of ROS-scavenging enzymes in transgenic plants indicate the presence of more non-water electron donors and an efficient removal of ROS respectively which in turn increases PSII efficiency; this is consistent with the chlorophyll-a fluorescence results, reported in this paper.

The performance of the photosynthetic apparatus is critical to the physiological status and vitality of plants under environmental stress. Measuring alterations of Chlorophyll-*a* fluorescence transients is now a widely applied technique for assessing performance of plants. Recent findings indicate that fluorescence rise from *F*_0_ to *F*_M_ indicates reduction of *Q*_A_ [58]. The so-called JIP-test, as explained by Strasser et al. [10,16,17,42] is the main explanatory model used for explaining OJIP transients [18,59,60]. Using this model, we have compared photosynthetic activities of stressed and control plants, and the test is a good, non-invasive tool for analyzing the effects of a variety of stress factors on plants [19,20,61,62]. In this study, OJIP transient curves changed in response to cold temperature as shown in **Fig 4A**. Further, the cold stress-induced changes in several JIP parameters were greater than that in *F*_V_/*F*_M_ (data not shown). Although *F*_V_/*F*_M_ is a well-known indicator of stress, many reports suggest that it is not as sensitive a parameter for analyzing temperature stress as it is for analyzing other abiotic stresses. Strauss et al. [6] screened several genotypes of *Glycine max* in response to chilling stress and reported that the *F*_V_/*F*_M_ ratio was much less sensitive and revealed very few differences between the genotypes. The OJIP transients were suggested to be more sensitive than *F*_V_/*F*_M_ for analyzing physiological damage to photosynthetic machinery under heat and chilling stresses [63]. Similar findings were reported in apple fruit [64] and tomato [65] where the *F*_V_/*F*_M_ remained similar even when the temperature difference between control and stressed samples was from 10 to 17°C.

Influence of cold stress on the photosynthetic apparatus, in particular on the donor side of PSII, is reflected in the OJIP curves (**Fig 4B**). A decrease in fluorescence slope at the J, I, and P steps indicates an impaired ET on the donor or the acceptor side of the PSII. This damage to the donor side of PSII has been corroborated with the K-band that appears at 300 μs and indicates intactness of the OEC [11,66]. A positive K-band indicates an increased slope of relative variable fluorescence at the origin, d*V*/d*t*_0_, which shows a maximal Δ*V*_OJ_ at about 300 μs. We recorded consistently positive K-bands in S599A lines (**Fig 4B)** suggesting an increased reduction rate of *Q*_A_ to *Q*_A_^−^, and the accessibility of non-water electron donors to the OEC. The L-band expresses the energetic connectivity (grouping) of the PSII components and this parameter demonstrated a marked difference in the slopes (**Fig 4C)**. Data on the L-band indicate that in NT plant samples, cold stress treatment results in a decrease of energetic connectivity, while the S599A plants showed an increase in energetic connectivity as evident by a negative L-band. Transgenic potato plants with reduced expression of a 33 kDa manganese stabilizing protein of PSII were reported to exhibit positive K-bands and negative L-bands [11]. Similarly, Yusuf et al. [43] reported positive K-bands and negative L-bands in transgenic *Vigna mungo* plants subjected to CdCl_2_ induced stress. More recently, positive K-bands and negative L-bands were also reported in perennial grasses showing resistance to heavy metal stress-induced by Zn and Cd [23].

The JIP-test represents translation of fluorescence data to biophysical parameters that quantify the energy flow through PSII [6]. The OJIP parameters, analyzed in order to determine the damage at the acceptor side of PSII also allows identifying critical parameters such as ABS, TR, ET, and RE [14]. Energy fluxes per RC express specific functional parameters, while the energy fluxes per excited CS are the corresponding phenomenological energy fluxes. The S599A plants under cold stress exhibited a significant decline in ABS/RC, ET_0_/RC, and TR_0_/RC from that under non-stressed conditions compared to those of PhyA and NT plants which showed a marginal decline in all three parameters (**Fig 5)**. Thus, higher average absorption, trapping, and ET in the S599A plants because of the inactivation of some RCs would mean that reduction in functional antenna size in cold stressed plants presumably enhanced energy transfer to active RCs compared to the NT and PhyA plants. This assumption is in line with a previous report suggesting that leaves under sun continuously regulate electron flux by reducing the PSII antenna size and by increasing controlled dissipation of energy [67]. A relatively increased value of TR_0_/RC of S599A to that of NT and PhyA plants under cold stress also indicates a stable association between energy absorbing chlorophyll molecules, in the form of active RC, and the trapping of this energy for efficient reduction of *Q*_A_ to *Q*_A_^−^. Under both normal and cold conditions, S599A plants showed the highest difference in the values of specific fluxes, followed by PhyA and NT (**Fig 5)**. These results indicate that under cold stress conditions, transgenic zoysiagrass plants were more efficient in the utilization of energy than the NT plants. Also, the increased phenomenological fluxes examined (ABS/CS, TR_0_/CS, and ET_0_/CS) of S599A plants under cold stress had a stimulating effect that resulted in a notable increase in the PI_ABS_ which incorporates the processes in the energy cascade from the first absorption events to plastoquinone reduction as well as a moderate increase in the PI_ABS_ and PI_total_ [68]. In contrast, the PI_ABS_ and PI_total_ of NT plants significantly decreased due to the cold-induced decreases in some parameters (**Fig 5)**.

**Fig 7:**
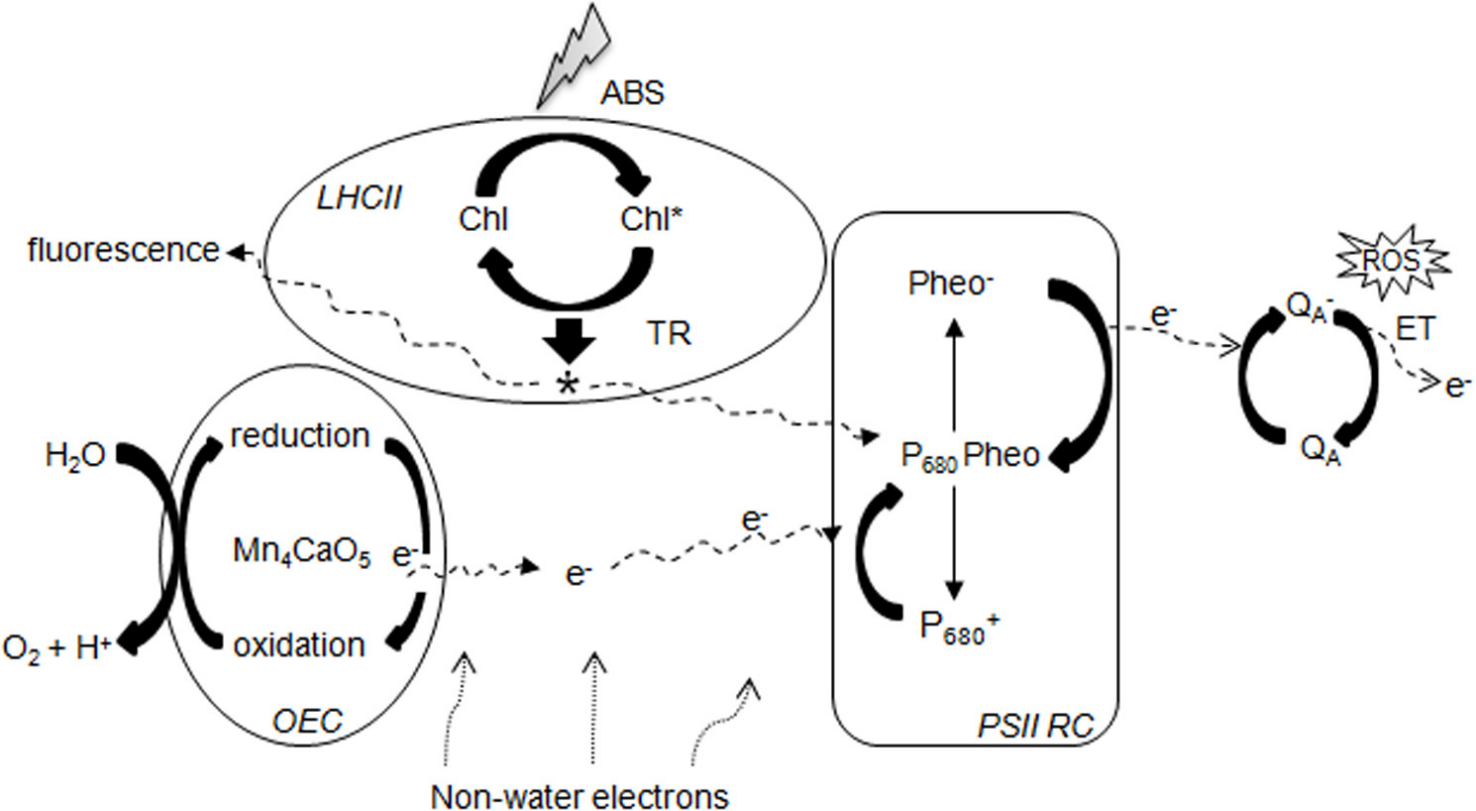
Hypothetical scheme of photosynthetic electron transport based on the emission kinetics of chlorophyll-a in cold stress treated zoysiagrass plants expressing mutant PhyA.

**Fig 7** illustrates the possible mechanism of cold stress-induced damage and potential sites of stress damage in zoysiagrass plants. During photosynthesis, light energy is converted into chemical energy in three consecutive basic steps, (i) ABS, absorption of photons by chlorophyll molecules in the light-harvesting complex (ii) TR, trapping of excitation energy by the RC, and (iii) ET, electron transport via PSII RCs. When plant tissues are exposed to a short pulse of strong light, chlorophyll-*a* absorbs light energy and uses it in photosynthesis (Chlorophyll-a*excited). At the same time, some light is emitted at a lower energy level as fluorescence. The first chemical step occurs when an excited donor pigment molecule with peak at 680 nm (P680) donates an electron to pheophytin (Ph), producing oxidized P680 (P680^+^) and reduced Ph (Ph^−^) in PSII [69]. The integrity of PSII after the primary reactions of photochemistry creates a primary redox potential within and around PSII between a primary electron donor pigment P680* and an electron acceptor, *Q*_A_. Besides producing ROS molecules such as H_2_O_2_, cold stress hampers the oxygen evolution at the OEC by decreasing the flow of electrons to the RCs of PSII thereby decreasing the concentration of *Q*_A_^−^. This decrease is reflected in the J slope of the NT as well as PhyA transgenic zoysiagrass plants (**Fig 4A**). A negative J slope under cold stress also means less accumulation of reduced electron carriers like plastoquinone and plastocyanin [61]. The impaired activity of OEC could be compensated by non-water electron donors for a short time. The fraction of electrons donated by water is lower in stressed samples. Under normal conditions, an intact manganese cluster (Mn_4_CaO_5_) at the OEC thus promotes electron donation from water to PSII-RC and restricts the entry of non-water electrons to the RC of PSII. Also, reduced phenomenological fluxes per active RC in NT, PhyA, and S599A plants under stress indicates the transfer of few electrons to the PSII-RC. Furthermore, reduced energy cooperativity (expressed by the L-bands) in NT plants indicates reduced cold stress tolerance in these plants compared to transgenic plants.

Taken together, our results indicate that Chlorophyll-*a* fluorescence analysis allows us to compare the physiological status of NT and PhyA transgenic zoysiagrass under cold stress versus control conditions. Our study shows that the expression of S599A induces a significant level of cold stress tolerance in zoysiagrass. Hence, we propose that this hyperactive mutant can be used to develop cold stress-resistant turfgrasses, although further studies are required prior to extending its application to other crops.
